# Risk Factors Related to New-Onset Diabetes after Renal Transplantation in Patients of a High Complexity University Hospital in Colombia, 20 Years of Experience

**DOI:** 10.1155/2020/8297192

**Published:** 2020-08-30

**Authors:** Guillermo E. Guzmán, Angela M. Victoria, Isabella Ramos, Alejandro Maldonado, Eliana Manzi, Juan F. Contreras-Valero, Liliana Mesa, Johanna Schweineberg, Juan G. Posada, Jorge I. Villegas, Luis A. Caicedo, Carlos E. Durán

**Affiliations:** ^1^Fundación Valle del Lili, Departamento de Endocrinología, Cra 98, No. 18-49, Cali 760032, Colombia; ^2^Universidad Icesi, Facultad de Ciencias de la Salud, Calle 18, No. 122-135, Cali, Colombia; ^3^Fundación Valle del Lili, Centro de Investigaciones Clínicas, Cra 98, No. 18-49, Cali 760032, Colombia; ^4^Fundación Valle del Lili, Departamento de Medicina Interna, Cra 98, No. 18-49, Cali 760032, Colombia; ^5^Fundación Valle del Lili, Departamento de Nefrología–Unidad de Trasplantes, Cra 98, No. 18-49, Cali 760032, Colombia

## Abstract

**Introduction:**

New-onset diabetes after transplantation (NODAT) is associated with immunosuppression. Its complications can negatively influence patients' quality of life, which is why it is important to study the associated risk factors and expand the possible therapies in this particular group of patients. *Materials and methods*. Case-control study nested in a retrospective cohort. It included patients who received kidney transplantation at the high complexity University Hospital Fundación Valle del Lili in Cali, Colombia, between 1995 and 2014. Two controls were assigned for each case, depending on the type of donor and the date of the surgery. Information was collected from clinical records and the institutional TRENAL registry. We carried out a descriptive analysis of the selected variables and identified the risk factors with conditional logistic regression.

**Results:**

122 cases were identified to which 224 controls were assigned. The median age was 44 years (IQR: 34–55), and 54% were men. Having >50 years of age at the time of transplantation (OR: 3.18, 95% CI: 1.6−6.3, *p* = 0.001), body mass index >30 kg/m^2^ (OR: 3.6, 95% CI: 1.3−9.7, *p* = 0.010) and being afro-descendant (OR: 2.74, 95% CI: 1.1−6.5, *p* = 0.023) were identified as risk factors for the development of NODAT. Pretransplant fasting plasma glucose >100 mg/dl (OR: 2.9, 95% CI: 1.4−6.4, *p* = 0.005) and serum triglycerides >200 mg/dl (OR: 2.5, 95% CI: 1.4−4.4, *p* = 0.002) were also reported as independent risk factors.

**Conclusion:**

We ratify some risk factors for the development of this important disease, which include certain modifiable characteristics. Interventions aimed at changes in lifestyle could be established in a timely manner before transplant surgery.

## 1. Introduction

Despite the new advances in surgical techniques, organ preservation, anesthesia, and immunosuppression, new-onset diabetes after transplantation (NODAT) remains as a frequent complication after a solid organ, bone marrow, or hematopoietic stem cell transplant [1]. According to the latest Standards of Medical Care in Diabetes developed by the American Diabetes Association (ADA), this entity refers to new-onset diabetes following transplant in a previously nondiabetic individual, excluding posttransplant hyperglycemia that resolves by the time of discharge [2, 3].

The incidence varies depending on the transplanted organ with an estimated value of up to 25–30% for renal transplantation, being the most frequently related solid organ [4, 5]. Additionally, these variations have been associated to factors such as multiple immunosuppressive treatments used, epidemiological characteristics of the receptor, and different definitions attributed to this diagnosis in the last years [6]. In Colombia, an incidence of 11% was documented for both liver and kidney transplantation. A higher incidence of this disease has been evidenced during the first six months after the transplant (around 20.5%), time in which patients are treated with higher doses of immunosuppressants [7, 8].

Different risk factors have been described for NODAT, including afro-descendant ethnicity, age over 45 years, family history of diabetes mellitus (DM), immunosuppressive therapy, and cytomegalovirus (CMV) infection, among others [6]. Previous in vitro studies with insulin-producing beta cells have identified multiple diabetogenic mechanisms of cyclosporine and tacrolimus, showing that both affect insulin secretion, decrease insulin content in beta cells, and affect insulin transcription [9]. In Colombia, the use of calcineurin inhibitors has been recognized as an important risk factor for NODAT, after smoking and having personal history of impaired fasting plasma glucose prior to hepatic transplantation [8].

NODAT constitutes an important public health problem since it triggers infectious and cardiovascular complications which can negatively influence the quality of life of patients leading to higher rates of graft rejection and short- or long-term mortality [10, 11]. Therefore, it is important to determine the risk factors related to this entity in our population to facilitate the development of appropriate prevention strategies.

## 2. Materials and Methods

Data from the TRENAL registry, which includes patients who receive kidney transplantation in the University Hospital Fundación Valle del Lili, was used to identify individuals with NODAT and their risk factors for the disease between 1995 and 2014 [7]. Based on the obtained results in this previous cohort study, we developed a nested case-control study to broaden the knowledge of risk factors that were not routinely included in TRENAL Registry. All patients with diagnosis of NODAT according to the ADA definition in the period described were defined as cases, and the controls were patients without NODAT at the time the case was identified [2]. Two controls were assigned to each case, depending on the type of donor (alive or deceased) and the date of transplant. From medical records, the following variables were reviewed: fasting plasma glucose and triglycerides levels prior to transplantation, as well as family history of DM.

Collected data were analyzed with Stata 12.1 (Stata Corporation, College Station, TX, USA). Quantitative variables were reported as medians and interquartile ranges (IQR). Categorical variables were described as frequencies and percentages. For comparison, the Wilcoxon rank-sum test was used for continuous variables and χ2 test for categorical variables. A conditional logistic regression model was used to evaluate associated factors with the development of NODAT. This study was approved by the FVL institutional review board.

## 3. Results

1126 patients were transplanted in the University Hospital Fundación Valle del Lili between 1995 and 2014. We identified 122 (11%) patients with NODAT to which 224 controls were assigned.  Table 1 shows the demographic and clinical characteristics of the studied population. Median age for all patients was 44 years (IQR 34–55), and 54% were male. Characteristics such as having >50 years of age at the time of transplantation (56% of cases vs 30% of controls, *p* < 0.0001), being afro-descendant (18% of cases vs 12.7% of controls, *p* = 0.006), having family history of DM (37% of cases vs 22% of controls, *p* = 0.002), and body mass index (BMI) >30 kg/m^2^ (13% of cases vs 3.7% of controls, *p* = 0.003), were statistically significant in their distribution among groups.

Table 2 shows the proportion of case and control patients who received treatment with steroids, tacrolimus, and mammalian target of rapamycin (mTOR) inhibitors, also the patients who received CMV prophylaxis prior to surgery, retransplantation, and who presented CMV infection. For these characteristics, there were no significant differences in its distribution between the case and control group.

After multivariable analysis, diverse characteristics were associated with the presentation of NODAT including age over 50 years at the time of transplantation (OR 3.18, 95% CI 1.6–6.3, *p* = 0.001) afro-descendant ethnicity (OR 2.74, 95% CI 1.1–6.5, *p* = 0.023), BMI >30 kg/m^2^ (OR 3.6, 95% CI 1.3–9.7, *p* = 0.010), fasting plasma glucose levels >100 mg/dl (OR 2.9, 95% CI 1.4–6.4, *p* = 0.005), and serum triglycerides >200 mg/dl (OR 2.5, 95% CI 1.4–4.4, *p* = 0.002) (Table 3).

## 4. Discussion

New-onset diabetes after transplantation is a metabolic complication that can result after a solid organ transplant. For renal transplantation, an incidence ranging from 4 to 30% has been reported [5, 12]. Our research team had previously reported a 5-year incidence of 9.1% [7]. In the present study, we demonstrate that NODAT development is associated with >50 years of age at the time of the surgery, afro-descendant ethnicity, BMI >30 kg/m^2^, hyperglycemia, and hypertriglyceridemia, risk factors that has also been previously reported by other authors.

The median age of our population was 44 years with 141 individuals with >50 years of age. Older age has been previously reported as a determining factor for the disease [12–15]. Kasiske et al. evidenced that transplant recipients between 45 and 59 years of age had a relative risk for NODAT of 1.9 (95% CI 1.73–2.09, *p* ≤ 0.0001) and with >60 years of age a relative risk of 2.6 (95% CI 2.32–2.92, *p* ≤ 0.0001) [13].

The association with afro-descendant ethnicity has also been described in other studies [16, 17]. Lima et al. reported it in their cohort study carried out in Brazil with 209 patients of whom 53 were afro-descendants. This characteristic showed a significant difference in its distribution between the group with NODAT and without the disease (30.2% vs 17.5%, *p* = 0.02), as it was seen in our study [17].

On the other hand, obesity has been linked to DM [18]. In patients with renal transplantation, obesity has been related to prolonged hospital stays and presentation of NODAT, a factor that has made renal transplantation in this population a controversial issue [19]. Despite the number of patients with obesity is increasing, the frequency of patients with this comorbidity in our study was low (6.8%), however, it was clearly associated with NODAT [20]. Therefore, considering options for weight reduction including bariatric surgery could be a strategy for prevention of metabolic complications such as NODAT [21].

Likewise, calcineurin inhibitors have been considered diabetogenic; specifically tacrolimus has been identified as an important factor in the development of NODAT [22]. The probable mechanism is through the inhibition of insulin secretion; nonetheless, in our study, bivariable analysis did not show this factor as statistically significant. This result could be explained by the methodological design of our study, since for the same population, it was previously identified as a risk factor for the disease [7].

Additionally, two viral infections have been linked to NODAT: hepatitis C virus, which could favor insulin resistance and generate a direct harmful effect on pancreatic cells, and CMV infection, which enhances cytokine-mediated pancreatic islet injury and apoptosis [23]. Nevertheless, in our study, these factors did not reach statistical significance in multivariable analysis.

Regarding pretransplant glycemic values, Cosio et al. have described that patients with values <90 mg/dl have a lower risk for NODAT (OR 0.46, *p* = 0.01), contrary to individuals with values between 101 and 110 mg/dl (OR 1.5, *p* ≤ 0.0001) and between 110 and 125 mg/dl (OR 7.6, *p* ≤ 0.0001) [24]. These values evidence a pretransplant insulin resistance, similar to findings reported by Gomes et al. recently [25].

With respect to pretransplant hypertriglyceridemia, its role as a marker for insulin resistance has been clearly studied, and in multivariable analysis of some retrospective cohorts, it has been associated with NODAT [26]. Porrini et al. in its cohort study showed that pretransplant triglyceride levels >200 mg/dl increase the risk of presenting this disease in patients who received treatment with tacrolimus (OR 3.26, 95% CI 1.4–7.8, *p* = 0.002), while this was not observed in those who received treatment with cyclosporine [27]. In our analysis, we found that, within the pretransplant laboratories, fasting plasma glucose >100 mg/dl (OR 2.9, 95% CI 1.4–6.4, *p* = 0.005) and serum triglycerides >200 mg/dl (OR 2.5, 95% CI 1.4–4.4, *p* = 0.002) are independent risk factors for the development of NODAT, which is consistent with previous findings in the literature.

There is relevant evidence that supports the association between family history of DM and the development of NODAT [6, 28]. This particular factor was significant in our bivariable analysis (OR 2.15, 95% CI 1.3–3.6, *p* = 0.003), but after multivariable analysis, its statistical significance was lost. This same phenomenon occurred in the retrospective study carried out by Sinangil et al. [26].

The main limitation of our study was that we developed our analysis based on a single health care center, and we carried out a retrospective collection of data which highlights possible memory and selection bias. Likewise, between the 20 years included in our study, there have been changes in clinical guidelines and protocols that may generate underreporting of some variables in certain periods of time.

## 5. Conclusions

NODAT is a common problem in our population and institution, which is a reference health care center for transplantation, especially renal transplantation. We ratify some risk factors for the development of this important disease, which includes modifiable ones that could be intervened in a timely manner.

## Figures and Tables

**Table 1:** Demographic and clinical characteristics of patients with renal transplantation between 1995 and 2014.

Characteristics	Total (*n* = 366)	Controls (*n* = 244)	Cases (*n* = 122)	*p* value
*Age, years*				
Median (IQR)	44 (34–55)	40 (30–52)	51 (40–60)	<0.0001
Age range	18–78	18–75	18–78	
Age >50 years, *n* (%)	141 (38)	73 (30)	68 (56)	<0.0001
Male gender, *n* (%)	196 (54)	126 (52)	70 (57)	0.299
Afro-descendant ethnicity, *n* (%)	53 (14.5)	31 (12.7)	22 (18)	0.006
Unknown, *n* (%)	11 (3)	3 (1.2)	8 (6.6)	

*BMI, kg/m* ^*2*^				
Median (IQR)	23.7 (21–26)	22 (20–24)	25.6 (23–28)	<0.0001
Weight range	15–40	15–40	18–40	
Unknown, *n* (%)	29 (8)	19 (7)	10 (8)	
BMI >30 kg/m^2^, *n* (%)	25 (6.8)	9 (3.7)	16 (13)	0.003
Family history of DM, *n* (%)	98 (27)	53 (22)	45 (37)	0.002

*Pretransplant fasting plasma glucose, mg/dl*				
Median (IQR)	88 (81–96)	86 (80–93)	92 (84–100)	<0.0001
Value range	23–205	23–119	65–205	
Pretransplant fasting plasma glucose >100 mg/dl, *n* (%)	57 (15.6)	23 (9)	34 (28)	<0.0001
Unknown, *n* (%)	3 (0.8)		3 (2)	

*Serum triglycerides, mg/dl*				
Median (IQR)	169 (114–240)	155 (107–221)	202 (140–298)	0.0001
Value range	34–1142	34–924	55–1142	
Unknown, *n* (%)	7 (1.9)	3 (1)	4 (3)	
Serum triglycerides >200 mg/dl, *n* (%)	137 (37)	75 (31)	62 (51)	<0.0001
Unknown, *n* (%)	7 (1.9)	3 (1)	4 (3)	
Pretransplant hepatitis C antibody test, *n* (%)	12 (3.28)	10 (4.1)	2 (1.6)	
Unknown, *n* (%)	4 (1.09)	1 (0.4)	3 (2.5)	0.099
Deceased organ donor, *n* (%)	311 (85)	208 (85)	103 (84)	0.836

IQR : interquartile range; BMI : body mass index; DM : diabetes mellitus.

**Table 2:** Risk factors for NODAT in patients with renal transplantation between 1995 and 2014.

Characteristics	Total (*n* = 366)	Controls (*n* = 244)	Cases (*n* = 122)	*p* value
Steroid treatment, *n* (%)	350 (96)	230 (94)	120 (98)	0.071
Treatment with tacrolimus, *n* (%)	148 (40.4)	103 (42)	45 (36)	0.328
Treatment with mTOR inhibitor, *n* (%)	59 (16)	33 (13)	26 (21)	0.056
CMV infection, *n* (%)	46 (12.5)	25 (10.25)	21 (17.2)	0.058
Prophylaxis for CMV, *n* (%)	100 (27)	67 (27)	33 (27)	0.934
Retransplant, *n* (%)	30 (8)	22 (9)	8 (6)	0.419

NODAT : new-onset diabetes after transplantation; mTOR inhibitor: mammalian target of rapamycin inhibitor; CMV: cytomegalovirus.

**Table 3:** Bivariable and multivariable analyses for risk factors associated with NODAT in patients with renal transplantation between 1995 and 2014.

Characteristics	Bivariable		Multivariable	
	OR (95% CI)	*p* value	OR (95% CI)	*p* value
Age >50 years	2.9 (1.8–4.7)	<0.0001	3.18 (1.6–6.3)	0.001
Male gender	1.27 (0.8–2.0)	0.312	—	—
Afro-descendant ethnicity	1.6 (0.87–2.9)	0.129	2.74 (1.1–6.5)	0.023
BMI >30 kg/m^2^	4.2 (1.7–10.0)	0.001	3.6 (1.3–9.7)	0.010
Family history of DM	2.15 (1.3–3.6)	0.003	1.7 (0.9–3.3)	0.098
Pretransplant fasting plasma glucose >100 mg/dl	3.82 (2.0–7.0)	<0.0001	2.9 (1.4–6.4)	0.005
Serum triglycerides >200 mg/dl	2.3 (1.4–3.6)	<0.0001	2.5 (1.4–4.4)	0.002
Pretransplant hepatitis C antibody test	0.4 (0.09–2.0)	0.302	—	—
Steroid treatment	3.7 (0.94–14.0)	0.060	3.8 (0.32–46)	0.286
Treatment with tacrolimus	0.69 (0.38–1.26)	0.236	—	—
Treatment with mTOR inhibitor	2.02 (1.0–3.8)	0.034	1.5 (0.55–4.1)	0.417
CMV infection	1.89 (1.01–3.5)	0.047	1.85 (0.7–4.9)	0.203
Prophylaxis for CMV	0.96 (0.49–1.8)	0.917	—	—
Retransplant	0.65 (0.29–1.43)	0.287	—	—

NODAT : new-onset diabetes after transplantation; BMI : body mass index; DM : diabetes mellitus; CMV: cytomegalovirus; OR: odds ratio; CI: confidence interval.

## Data Availability

The data used to support the findings of this study are restricted by Fundación Valle del Lili Ethics Committee in order to protect patient privacy. Data are available from Dr. Guillermo E. Guzmán for researchers who meet the criteria for access to confidential data.
